# Identification and characterisation of a novel anti-viral peptide against avian influenza virus H9N2

**DOI:** 10.1186/1743-422X-6-74

**Published:** 2009-06-05

**Authors:** Mohamed Rajik, Fatemeh Jahanshiri, Abdul Rahman Omar, Aini Ideris, Sharifah Syed Hassan, Khatijah Yusoff

**Affiliations:** 1 Department of Microbiology, Faculty of Biotechnology and Biomolecular Sciences, University Putra Malaysia, UPM Serdang, Selangor, 43400, Malaysia; 2 Institute of Bioscience, University Putra Malaysia, UPM Serdang, Selangor, 43400, Malaysia; 3 Faculty of Veterinary Medicine, Universiti Putra Malaysia, UPM Serdang, Selangor, 43400, Malaysia; 4 School of Medicine and Health Sciences, Monash University, Sunway Campus, Kuala Lumpur, Malaysia

## Abstract

**Background:**

Avian influenza viruses (AIV) cause high morbidity and mortality among the poultry worldwide. Their highly mutative nature often results in the emergence of drug resistant strains, which have the potential of causing a pandemic. The virus has two immunologically important glycoproteins, hemagglutinin (HA), neuraminidase (NA), and one ion channel protein M2 which are the most important targets for drug discovery, on its surface. In order to identify a peptide-based virus inhibitor against any of these surface proteins, a disulfide constrained heptapeptide phage display library was biopanned against purified AIV sub-type H9N2 virus particles.

**Results:**

After four rounds of panning, four different fusion phages were identified. Among the four, the phage displaying the peptide NDFRSKT possessed good anti-viral properties *in vitro *and *in ovo*. Further, this peptide inhibited the hemagglutination activity of the viruses but showed very little and no effect on neuraminidase and hemolytic activities respectively. The phage-antibody competition assay proved that the peptide competed with anti-influenza H9N2 antibodies for the binding sites. Based on yeast two-hybrid assay, we observed that the peptide inhibited the viral replication by interacting with the HA protein and this observation was further confirmed by co-immunoprecipitation.

**Conclusion:**

Our findings show that we have successfully identified a novel antiviral peptide against avian influenza virus H9N2 which act by binding with the hemagglutination protein of the virus. The broad spectrum activity of the peptide molecule against various subtypes of the avian and human influenza viruses and its comparative efficiency against currently available anti-influenza drugs are yet to be explored.

## Background

Avian influenza A viruses (AIV) are enveloped, segmented and negative-stranded RNA viruses, that circulate worldwide and cause one of the most serious avian diseases called Bird Flu, with severe economic losses to the poultry industry [1]. They are divided into different subtypes based on two surface glycoproteins, hemagglutinin (HA) and neuraminidase (NA). Currently, there are 16 different types of HA and nine different types of NA circulating among aquatic birds [2]. Although wild birds and domestic waterfowls are considered natural reservoirs for all subtypes, they usually do not show any symptoms of the disease. Domestic birds such as chickens are main victims of this virus especially of H5, H7 and H9 subtypes. The H9N2 viruses are endemic and highly prevalent in poultry of many Eurasian countries. These viruses cause severe morbidity and mortality in poultry as a result of co-infection with other pathogens [3,4]. Recent studies have also shown that H9N2 prevalence in poultry pose a significant threat to humans [5-8].

Adamantane derivatives (amantadine and rimantadine) and neuraminidase inhibitors (NAIs; zanamivir and oseltamivir) are currently used for the chemoprophylaxis and treatment of influenza [9]. The drugs should be administered within 48 hours of infection to get the optimum results. Amantadine binds to and blocks the M2 ion channel proteins function and thereby inhibits viral replication within infected cells [10]. NAIs inhibit the activity of neuraminidase enzymes and thus prevent the exit of virus from the infected cells [11].

In the last 15 years, the rate of amantadine resistant strains has risen from 2% during 1995 – 2000 to an alarming 92.3% in the early 2006 in the United States alone for the H3N2 subtype [12] although none of the neuraminidase inhibitors and adamantane resistant H5N1 viruses were reported in the south east asian region from 2004 to 2006 [13]. Usually, these viruses are highly pathogenic and transmissible among animals [14,15]. The viruses resistant to these drugs emerge due to mutations either at active sites of NA, altering its sensitivity to inhibition, or a mutation in the HA [9]. The mutations at HA reduce the affinity of the proteins to the cellular receptors and enable the virus to escape from infected cells without the need of NA. In several instances, strains which were resistant to both classes of antiviral drugs have been isolated from patients [16-18]. For these reasons, it has become necessary to identify novel drugs against the virus to control and treat infections.

Traditionally, the generation of new drugs involves screening hundreds of thousands of components against desired targets via *in vitro *screening and appropriate *in vivo *activity assays. Currently, new library methodologies have been developed with alternative and powerful strategies, which allow screening billions of components with a fast selection procedure to identify most interesting lead candidates. In this present study we used one of such methodologies called phage display technology to select novel peptides against avian influenza virus H9N2. The selected peptides were characterised for their anti-viral properties and their interaction site with the virus was identified by yeast two-hybrid assay and co-immunoprecipitation. The results showed that one of the peptides possesses good anti-viral property and inhibits the viral replication by binding with HA protein. The broad range anti-viral activity of the peptide against various subtypes of the virus is yet to be studied and if it turned positive, the peptide may serve as an alternative anti-viral agent to replace current potentially inefficient drugs.

## Results

### Selection of peptides that interact with AIV

Peptides selected from phage display library have been used as effective anti-microbial agents in previous studies [19]. In this study, a *7-mer *constrained phage displayed random peptide library containing about 3.7 × 10^9 ^different recombinant bacteriophages were used to select ligands that interact with the purified target molecule, AIV subtype H9N2. Four rounds of panning were carried out, each with a slight increase in stringency to isolate high-affinity peptide ligands.

Table 1 shows the heptapeptide sequences obtained from four rounds of panning the peptide library against AIV subtype H9N2. Seventeen out of 35 phages analysed from the fourth round represented the sequence NDFRSKT and other major sequences found in the final round of panning were LPYAAKH and ILGDKVG. A new sequence carrying the peptide QHSTKWF emerged during the fourth round of panning represented 10% of the total phages sequenced.

**Table 1:** Heptapeptides binding to AIV subtype H9N2 and streptavidin selected from the phage display random peptide library.

Rounds of panning	Heptapeptide sequences	Frequency of sequences (%)
4^th ^round	**NDFRSKT**	47
	QHSTKWF	10.5
	LPYAAKH	5
	ILGDKVG	5
	Unrelated sequences	23
		
**Panning of Streptavidin**		
3^rd ^round Streptavidin	**HPQ**FLSL	55
	GLYN**HPQ**	27
	Unrelated sequences	18

After 4 rounds of selection and amplification 20, 35 and 35 individual clones from the 2^nd^, 3^rd ^and 4^th ^rounds, respectively, were sequenced for AIV whereas 20 clones were sequenced from the 3^rd ^round of panning against Streptavidin.

Biopanning of the phage library against streptavidin (the positive control) gave a consensus sequences containing HPQ motif which totally represented 82% of the total phages screened from the third round of panning and these results are in good agreement to the reported findings [20-22]. No recognisable consensus sequence was observed with BSA, which served as a negative control. The peptide NDFRSKT was named as P1 (C-P1 – cyclic form; L-P1 – Linear form; FP-P1 – fusion phage displaying this peptide).

### Estimation of binding abilities of selected phage clones

**Figure 1:**
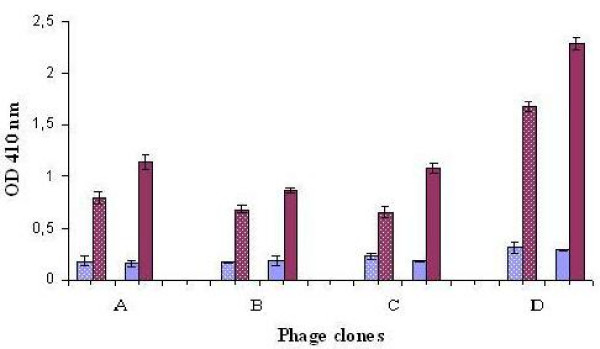
**Binding ability of all four recombinant phages to AIV H9N2**. Briefly, viruses were coated in the microwell plate at two different concentrations (5 μg and 10 μg/ml; 200 μl) and were detected by two different concentrations of recombinant phage molecules (10^12 ^pfu/ml and 10^11 ^pfu/ml). Dotted bars represent the 5 μg of target whereas solid bars represent 10 μg of target. All the four types of recombinant phage particles could able to detect the target AIV. Wild type phage M13 was used as control (Data not shown to avoid complexity of the graph). A – ILGDKVG (5%), B – NDFRSKT (47%), C – LPYAAKH (23%), D – QHSTKWF (5%), Blue Square – 10^11 ^pfu/ml, Grey Square-10^12 ^pfu/ml

Recombinant phages selected from the fourth round of panning were further analysed for their binding specificity by phage-ELISA which was carried out with all the four recombinant phages in varying phage concentrations against two different virus concentrations (5 μg and 10 μg/100 μl). The results (Figure 1) showed that all the phages selected from the biopanning were able to bind the virus efficiently and the higher the concentration of the recombinant phages, the higher the signal irrespective of the concentration of the virus.

### Antiviral activity of peptides and fusion phages in vitro

**Figure 2:**
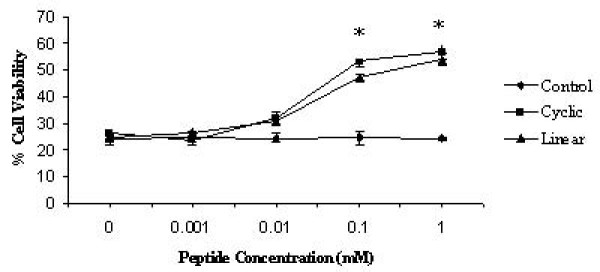
**Antiviral activity of peptides in vitro**. MDCK cells were inoculated with untreated AIV H9N2 or treated with increasing concentration of linear, cyclic and control peptides and the cell viability was determined by MTT assay. Results shown are the mean of three trials +/- SD. (*, statistical significance (P < 0.05)

**Figure 3:**
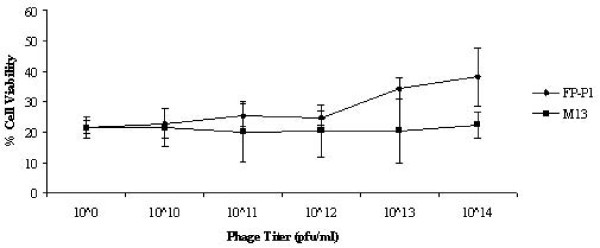
**Antiviral activity of fusion phages in vitro**. MDCK cells were inoculated with untreated virus (AIV H9N2) or virus treated with increasing concentration of fusion phages and the cell viability was determined by MTT assay. Results shown are the mean of three trials +/- SD.

The fusion phage FP-P1 and the cyclic as well as linear peptides were evaluated for its ability to inhibit viral-induced cell death using a cytotoxicity assay as explained by Jones et al (2005). Briefly, MDCK cells were mock inoculated (medium alone) or inoculated with different concentrations of phage or peptide treated AIV virus (MOI of 0.05 pfu/cell), and cell viability was evaluated at 48 hpi. If the FP-P1 phages were able to inactivate the AIV, then the AIV might not be able to induce the cell death and so the viability will increase. Interestingly, pre-treatment with increasing concentration of FP-P1 as well as the peptides increased the cell viability in dose dependent manner. More than 100% increase in viability was observed with the fusion phage and peptide treatment. In contrast, treatment with the wild type phage and control peptides did not show any significant increase in viability (Figure 2 and 3). This observation demonstrates that the fusion phage FP-P1 as well as the peptides (both in linear as well as cyclic form) was capable of inactivating the virus or inhibiting the viral replication *in vitro*.

### Antiviral activity of peptides and fusion phages in ovo

**Figure 4:**
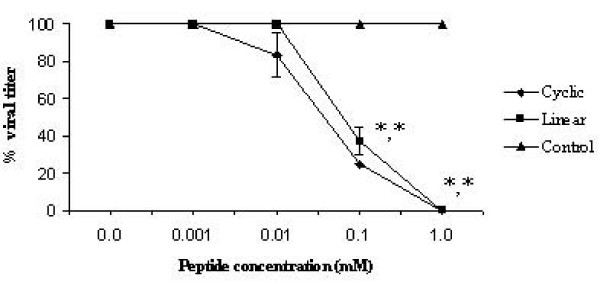
**Antiviral activity of peptides in ovo**. The peptide concentration needed to inhibit 50% of the virus growth was determined using different concentrations of peptides. Experiments were done in triplicates and the error bars represent the standard error of the mean. *, statistical significance (P < 0.05) (The SEM value is not shown for other values as there was little variation between repeated experiments).

Peptides were evaluated for their antiviral activity *in ovo *against AIV H9N2. Briefly, different concentrations of both cyclic and linear peptides (0.00, 0.001, 0.01, 0.1 and 1 mM) were mixed with constant amount of virus (8 HAU) and injected into allantoic cavity of embryonated chicken eggs. After 3 days, the allantoic fluid was harvested and the HA titer was determined. Complete inhibition was observed at the concentration 1 mM (Figure 4). The IC_50 _values of both cyclic and linear peptides were 48 μM and 71 μM respectively.

**Figure 5:**
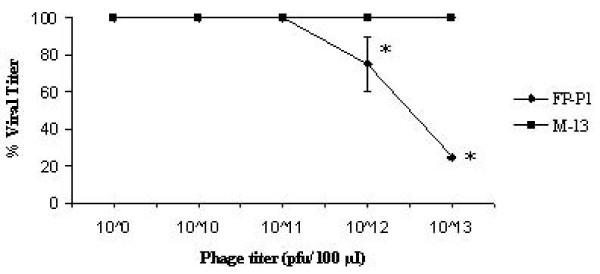
**Antiviral activity of fusion phages in ovo**. The fusion phage concentration needed to inhibit 50% of the virus growth was determined using different concentrations of recombinant phages FP-P1. Experiments were done in triplicates and the error bars represent the standard error of the mean. *, statistical significance (P < 0.05) (The SEM value is not shown for some data as there was no variation between repeated experiments).

To evaluate the efficacy of the fusion phage to inhibit the virus propagation *in ovo*, different pfu (10^8 ^– 10^13^/100 μl) of recombinant fusion phages were mixed with constant amount of virus (16 HAU) and injected into the allantoic cavity of embryonated chicken eggs. After 3 days, the allantoic fluid was harvested and the HA titer was measured. The fusion phage FP-P1 reduced the viral titer in the allantoic fluid upto 4 fold at the concentration more than 10^13 ^pfu/100 μl (Figure 5). Based on the dose response curve, the IC_50 _for FP-P1 was approximately 5 × 10^11 ^pfu/100 μl.

**Figure 6:**
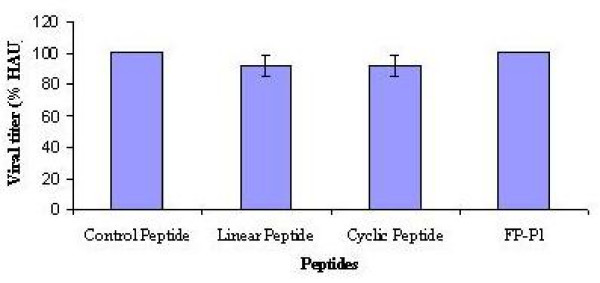
**Effect of peptides against NDV**. Cyclic, linear and FP-P1 at 100 μM concentrations were analysed for their inhibitory ability against NDV in embryonated chicken eggs. Viral titers in the allantoic fluid were measured as HA units. Results are shown as the mean of three independent experiments and error bars represent the standard deviation of the mean. None of the peptides showed a statistically significant result (ANOVA, p = 0.596).

Besides, to determine whether these peptides inhibit the virus replication specifically, these peptides (linear, cyclic and FP-P1) were tested for inhibitory effects against NDV strain AF2240. None of these molecules do not posses significant (ANOVA, p = 0.596) inhibitory effect against NDV replication (Figure 6).

### Inhibitory effects of peptides and fusion phages on virus adsorption onto chicken red blood cells (cRBCs)

Influenza A viruses, including AIV sub-type H9N2, have the ability to adsorb onto chicken RBCs, resulting in hemagglutination. So, inhibition of agglutination of blood cells was used to test the hypothesis that peptides C-P1, L-P1 and fusion phage FP-P1 inhibited viral attachment. Initially, the inhibition of viral-induced agglutination of cRBCs by the peptides and fusion phages were monitored. Twofold dilutions of untreated or peptide/phage treated virus were incubated with cRBCs, and agglutination was observed. All the three forms of peptides completely inhibited AIV sub-type H9N2 agglutination in a dose-dependent manner at concentrations of 100 μM or more (Table 2). In contrast, the control peptide CSWGEYDMC had no effect on agglutination.

**Table 2:** Inhibitory ability of the cyclic and linear peptides against the hemagglutination activity of the avian influenza virus H9N2.

**Inhibitory Molecule**	Minimum Inhibitory Concentration*
Cyclic Peptide	100 μM
	
Linear Peptide	100 μM
	
Fusion Phage	10^13 ^pfu/100 μl

* Minimum concentration of peptides or phage required to inhibit the hemagglutination activity of 32 HAU of AIV

### Inhibitory effects of peptides and fusion phages on neuraminidase activity

Based on the ability of the peptides and fusion phage to inhibit viral attachment, we hypothesised that the peptide interacted either with NA or HA since changes to either surface glycoproteins can alter fitness of the virus. Moreover, the biopanning experiment was carried out against the whole virus. As NA is one of the most abundant surface glycoproteins, the chances for binding of the peptides to this protein are relatively high. To determine if peptides or fusion phage inhibited enzymatic activity, untreated or peptide/fusion-phage – treated virus was tested for enzymatic activity. Untreated and cyclic peptide or fusion phage treated virus had similar enzymatic activity, suggesting that both of them had no effect on NA activity. But linear peptide showed reduced Neuraminidase activity at very very high concentrations. 1000 μM or more concentration of the linear peptide was required to reduce around 35% of the enzyme activity (data not shown). Considering the inability of cyclic and FP-P1 to inhibit the NA activity and the very limited ability of linear peptide it can be deduced that the linear peptide may non-specifically interact with the NA protein, perhaps taking advantage of its flexible nature.

### Inhibition of phage binding to AIV by antibody

**Figure 7:**
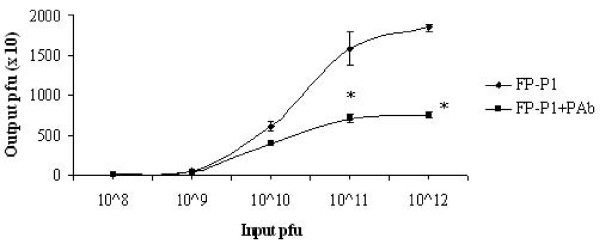
**Antibody-phage competition assay**. The phage competes with polyclonal antibodies for binding site on AIV, suggesting they may share common binding sites. Experiments were done in triplicates and the error bars represent the standard deviation of the mean. *, statistical significance (P < 0.05)

Polyclonal antibody (pAb) and phage competition assay was performed to understand whether they both share common binding sites. Briefly, either fusion phages alone or fusion phage-antibody mixtures were added into wells coated with the virus and the eluted phages were titered. Figure 7 demonstrates that the fusion phages FP-P1 were able to compete with the pAb for binding sites on AIV. In the presence of the antibody, the number of phages bound to the AIV coated wells reduced dramatically as a result of the competition between these two molecules for the same binding site on AIV. For example, at input pfu of 1 × 10^12^/100 μl, the output pfu for the FP-P1 phage alone was 1.8 × 10^4 ^plaques but in the presence of pAb, the output was reduced to 7.5 × 10^3 ^plaques, which is almost 2.4-fold reduction. This result clearly shows us that the phage molecules that display peptides on their surface can compete for the epitope binding sites on AIV with polyclonal antibodies.

### Peptide-phage competition assay

**Figure 8:**
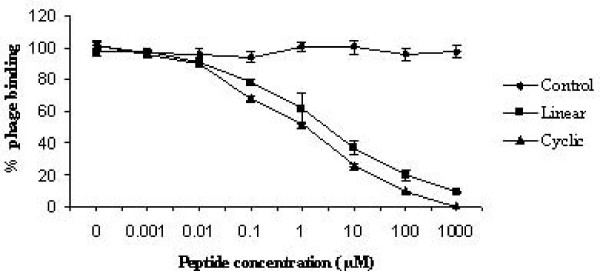
**Peptide-phage competition assay**. The peptides competes with the fusion phage FP-P1 for binding sites on AIV, suggesting that peptides displayed on the fusion phage FP-P1, and not other parts of the phage, binds to the AIV. Experiments were performed in triplicates and the error bars represent the standard deviation of the mean.

In order to identify whether the synthetic peptides and the phages (FP-P1) compete for the same binding sites on AIV H9N2, a peptide-phage competitive assay was performed. When the peptides (both linear and cyclic) were pre-incubated with the virus, the number of phages bound to the virus was reduced gradually in a dose-dependent manner. At 1 mM concentration of the peptides, the phage binding was almost completely inhibited (Figure 8). The control peptide does not possess any inhibitory effects on phage binding to AIV.

### Interaction between C-P1 peptide and HA_t_/NA protein by yeast two hybrid assay

The yeast two-hybrid assay was employed to validate the HA-P1 interaction and also to identify any interaction between NA-P1. To eliminate the false positive results (the possibility of Binding Domain (BD)-P1, Activation Domain (AD)-HA_t _and AD-NA fusion proteins themselves bringing about activation of the reporter genes), various combinations of the recombinant plasmids along with the parental vector were co-transformed into the yeast competent cells (Table 3). Three independent clones from each co-transformation were analysed for the activation of the β-galactosidase (β-gal) reporter genes. As shown in Table 3, the co-transformed parental vectors did not show any β-gal activities. When BD-P1 and AD-HA_t _or BD-P1 and AD-NA fusion constructs were co-transformed separately along with their respective parental vectors, no β-gal activity was detected either. The co-transformed BD-P1 and AD-HA_t _as well as BD-P1 and AD-NA showed comparatively high level of β-gal activity (25 and 3.5 Miller Units respectively). This observation showed that the P1 peptide bind with both with HA glycoprotein as well as the NA glycoprotein. The P1 interaction with HA glycoprotein support the previous experimental observation of hemagglutination inhibition. As the yeast two-hybrid assay provided ambiguous result regarding the NA-P1 interaction, further experimental analysis (co-immunoprecipitation) was carried out.

**Table 3:** P1: HA_t_/NA interactions in the yeast two-hybrid system

DBD Vectors^a^	AD Vectors^a^	β-gal activity^b^
**Background**		
BD	AD	0.05
BD-P1	AD	0.07
BD	AD-HA_t_	0.05
BD	AD-NA	0.03
		
**P1:HA**_t_**/NA interactions**		
BD-P1	AD-HA_t_	25
BD-P1	AD-NA	3.5

^a^pHyblex/Zeo and pYESTryp2 are vectors encoding the LexA DNA binding domain (BD) and B42 transcriptional activation domain (AD), respectively.

^b^Average β-gal activity (Miller Units) of 3 independent colonies for each co-transformation (The SD value is not shown as there was very little variation between repeated experiment).

### HA_t_-P1, NA-P1 interaction study by Co-immunoprecipitation

**Figure 9:**
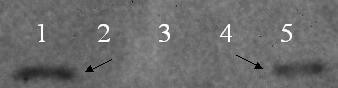
**Western blot analysis of immunoprecipitated HA_t_-P1 complex**. *In vitro *translated NA protein or HA_t _protein was mixed with P1 peptide and the complex was co-immunoprecipitated using anti-AIV serum and the eluted complex was analysed by SDS-15% PAGE, electrotransferred to a nitrocellulose membrane and probed with anti-His monoclonal antibody (Novagen, USA). Lane 1: HA_t _and P1 complex; lane 2: NA and P1 complex; For control, in vitro translated NA or HA_t _mixed with control peptide SWGEYDM and detected using anti-His antibodies. Lane 3: HA_t _and Control peptide complex; Lane 4: NA and control peptide complex; Lane 5: *in vitro *translated P1 peptide (~12 kDa). The arrow indicates the precipitated P1 protein in the HA_t_-P1 complex and the *in vitro *translated P1 peptide.

In order to verify the binding ability of the peptide P1 with HA_t _and NA proteins through Co-IP method, these three proteins were initially synthesised by in vitro transcription and translation methods. The P1 peptide was mixed either with the HA_t _protein or NA protein separately to allow the binding overnight or after incubation, the HA or NA protein present in the mixture was immunoprecipitated by anti-AIV polyclonal serum. After three rounds of washing, the bound P1 was detected by anti-His monoclonal antibodies (Novagen, USA). The P1 peptide was detected only in the HA complex (Figure 9). There was no P1 peptide visible in the NA complex (data not shown). This experiment confirmed the interaction of the P1 peptide to the HA protein.

### Peptide Toxicity

**Figure 10:**
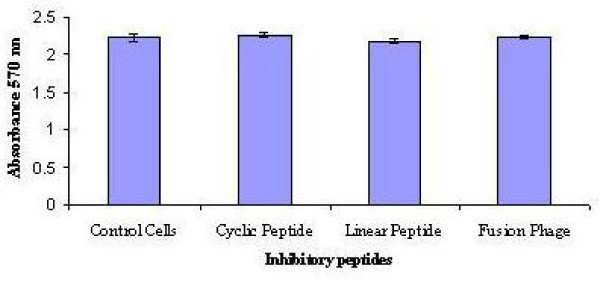
**In vitro toxicity of inhibitory peptides**. MDCK cells were treated with 100 μM of C-P1 or L-P1 or 10^14 ^pfu/ml of FP-P1 and the cell viability was analysed by MTT assay after 24 hrs of incubation (mean of three experiments +/- SD). No statistically significant differences in cell viability were observed (Students t test, P > 0.05).

To analyse the cellular toxicity properties of the peptides and fusion phages, MDCK cells were exposed to 100 μM of cyclic, linear peptides or 10^13 ^pfu/100 μl of FP-P1 for 24 hrs and the cell viability was determined by MTT assay. There was no significant difference (Students t test, P > 0.05) observed in the cell viability of control and peptide treated cells (Figure 10).

## Discussion

Emerging and re-emerging infectious diseases remain to be one of the major causes of death worldwide. The current outbreak of avian influenza viruses is a major global concern due to the increasing number of fatalities among the poultry as well as human cases. Its highly mutative nature makes the current antiviral drugs not very effective. Therefore, there has been a constant need for broad-spectrum antiviral drugs against the currently circulating human as well as avian strains.

In this study, a phage displayed peptide library was used to select anti-viral peptides against the AIV H9N2. At the end of biopanning, four different peptide sequences were identified. Matching of these peptide sequences with protein sequences in the protein data banks (Swiss Prot and NCBI) showed no significant homology with any protein sequences. It is possible that these peptides might mimic a discontinuous binding site in which amino acids are brought from different positions of a protein to form an essential contact area with the virion [23,24]. The lack of antiviral activity by the control peptide as well as the wild type phage suggests that the antiviral property of the peptides is specific to those peptides and neither a general property of any oligomeric peptide or wild type M13 bacteriophages nor based on charge or hydrophobic interactions. The peptide phage competition assay proved that the peptide displayed on the phage surface not the other parts of the phage binds to the virus.

Among the four different fusion phages isolated from the phage display library, the phage displaying the sequence NDFRSKT was selected for further analysis as it represented highest number of clones in the final round of biopanning. Besides, the peptides LPYAAKH, ILGDKVG, and QHSTKWF showed negligible or no anti-viral activity (data not shown); therefore, no further analyses on these peptides were carried out.

The *in ovo *model has been previously employed successfully by our group Ramanujam *et al*. [25] and Song *et al*. [26] to study the inhibitory effect of anti-viral molecules against the Newcastle disease virus and influenza virus respectively. Therefore, the antiviral activity of the synthetic peptides and the fusion phages themselves (or simply denoted as inhibitory peptides hereafter) were investigated in embryonated chicken eggs. All the peptides showed good anti-viral properties against AIV and interestingly there was no significant anti-viral effect found against NDV strain AF2240. Pre-treatment with the peptides or fusion phages reduced the AIV titre manifold (from 2 fold to 6 fold based on the type of peptide and number of days of treatment) in the infected allantoic fluid. But the post-infection treatment failed to protect the embryo (data not shown). However, it should be noted that the peptide was injected only once in the study and besides, the amino acids of the peptide were of L-isomers which are more prone to protease degradation inside the allantoic cavity.

Nevertheless, both cyclic and linear forms of peptides as well as the fusion phages proved their worth as antiviral molecules in varied potential levels. Among them, the cyclic peptide possessing the sequence CNDFRSKTC showed higher antiviral properties. The reason maybe its small size (only 9 amino acids in length for cyclic peptide) which helps its easy access to the respective binding site on the target molecule. Moreover, the cyclic peptides possess a stable structure due to the disulfide bond formed between the flanked cysteine residues which help to attain a stable interaction at a short time when compared to the linear peptides [27,28]. Small peptide molecules have been used in the development of peptide based vaccines for melanoma [29], inhibitors against HIV [30], Dengue and West nile virus [31] and anti-angiogenic in the treatment of angiogenesis related diseases [32].

As whole virus particles were used in biopanning experiments, in principle, the selected peptides might interact with any of the three surface proteins such as HA, NA and M2. Since these inhibitory peptides possess strong anti-viral activity when used at pre-infection not at post-infection and also inhibit the hemagglutination, it can be deduced that the peptides (NDFRSKT and CNDFRSKTC) prevent the viral replication by inhibiting the attachment or entry of the virus into the target cells. There are many studies on the targeting of the conserved region of the HA protein. Recently, Jones *et al. *[33] identified that a well known cell-penetrating peptide, derived from the fibroblast growth factor 4 (FGF-4) signal sequence, possesses the broad-spectrum anti-influenza activity, which act by blocking the entry of virus through the HA protein interaction.

Neuraminidase (NA) is the second most abundant surface protein and responsible for the neuraminidase activity of the virus. It is important both for its biological activity in removing sialic acid from glycoproteins and as a major antigenic determinant that undergoes variation. At present, the neuraminidase inhibitors such as zanamivir and oseltamivir are preferentially used for the treatment and prophylaxis of influenza [9], as the NA protein is less mutative when compared with HA. There are three receptor binding sites, two at the distal ends of both HA subunits and the third one in the NA protein [34] and changes in both HA and NA glycoproteins will affect the fitness of the virus [35]; therefore, the effect of peptide on the neuraminidase protein was assessed. Unfortunately, this experiment showed a negative result for the fusion phages and cyclic peptides and partial inhibition result at very high concentration of linear peptide (~35% inhibition at 1000 μM). The latter inhibition may be nonspecific due to the increased ability of the linear molecules to attain a structure that facilitates the binding with NA molecule or merely based on hydrophobicity and charge.

The HA-P1 and NA-P1 interaction was further analysed by the yeast two-hybrid system and co-immunoprecipitation. There has been a problem in amplifying the full length clone of *HA *gene for the past few years in our laboratory. The same problem has also been reported in few other laboratories working with the same strain in this region. The 3' end of the vRNA could not be amplified either by primer designed for conserved region or gene specific region based on other similar strain's sequence. The HA protein should be cleaved into two disulfide linked HA_1 _and HA_2 _in order to be infectious. The C-terminal HA_2 _region is very important as it accounts for the entry of the virus into the host cell and thus serves as a fusion protein [36]. Therefore, the truncated HA protein representing C-terminal end (278 aminoacids) of the full length HA protein was used for the yeast two-hybrid and co-immunoprecipitation experiments. The yeast hybrid assay turned positive for the both HA and NA proteins although the β-galactosidase activity for HA is nearly 7 fold higher than the NA. Although, there was negligible or no interaction between NA and P1 as per the results of NA inhibition test and co-immunoprecipitation results, the yeast two-hybrid experiment showed a significant NA-P1 interaction which is almost 100 times higher than the control. So, NA-P1 interaction cannot be simply ignored and further investigations are required to analyse the kind of interaction between the NA glycoproteins and peptide P1. But, the HA and P1 interaction has been clearly demonstrated without any doubt in all the performed experiments.

## Conclusion

Taking all together, this study has identified a novel antiviral molecule which inhibits the avian influenza virus infection by interacting with the surface glycoprotein HA and preventing its attachment to the host cell. To our knowledge, the selected peptide is the only antiviral peptide amongst the currently identified anti-viral peptides with 7 or 9 amino acids in length. This short sequence will be an added advantage for commercialisation purpose as it can greatly reduce the cost of production. However, additional studies are required to define the broad-spectrum activity of the peptide against various strains including the currently circulating potential pandemic strains such as H1N1 and H5N1 as well as its diagnostic potential.

## Methods

### Viruses, Cells and viral purification

Avian influenza A/Chicken/Iran/16/2000(H9N2), a low pathogenic avian influenza virus and Newcastle disease virus (NDV) strain AF2240 was kindly provided by Abdul Rahman Omar. Viruses were propagated in 9-day old specific pathogen free embryonated chicken eggs. The allantoic fluid was clarified and the viruses were purified and concentrated as explained previously [25]. The virus titer was determined by hemagglutination test (HA) and the protein concentration of the purified virus was determined by Bradford assay [37].

### Selection of peptides against AIV sub-type H9N2

The virus (15 μg/ml; 100 μl) was coated onto a microtiter plate well with NaHCO_3 _(0.1 M, pH 8.6) buffer overnight at 4°C. Streptavidin (0.1 mg/ml; 100 μl) was also coated and used as positive control. Phages from a disulfide constrained 7*-mer *phage display random peptide library (New England Biolabs, USA) were biopanned as explained by the manufacturer. The amplified phages from the first round of biopanning were used for the second round of biopanning. Totally four rounds of biopanning were carried out. Phage titration was carried out according to the method described by Sambrook *et al *[38]. Phages were propagated in *Escherichia coli *(*E. coli*) host cells grown in LB broth (1 L). The phage particles were precipitated by PEG and purified through cesium chloride density gradient centrifugation as descried by Smith and Scott [39].

### Sequence analysis of phagemids

The nucleotide sequence encoding the hypervariable heptapeptide region of pIII coat protein of M13 phage was sequenced by 1^st ^Base Laboratories Sdn Bhd, Kuala Lumpur, with the -96 gIII sequencing primer 5' CCC TCA TAG TTA GCG TAA CG 3'. Sequence analyses such as comparison with wild type M13 phage pIII coat protein and prediction of amino acid sequences were performed with the free bioinformatics software package, SDSC biology workbench 3.2.

### Estimation of binding abilities of selected phages

The avian influenza viruses were coated (5 or 10 μg/ml; 200 μl) on a microtiter plate with TBS buffer overnight at 4°C. The excess target was removed and blocked with blocking buffer (milk diluent KPL, USA) for 2 h at 4°C. The plate was then washed with 1× TBST (TBS and 0.5% [v/v] Tween 20). Selected phages were added into the well at the concentration of either 10^12 ^pfu/ml or 10^11 ^pfu/ml and incubated for 2 h at room temperature. The plate was again washed 6 times with 1× TBST. HRP-conjugated anti-M13 antibody (Pharmacia, USA) was diluted into 1:5000 with blocking buffer and added 200 μl into each well, incubated at room temperature for 1 h with agitation. It was then washed 6 times with 1 × TBST as explained above. 200 μl substrate solution (22 mg ABTS in 100 ml of 50 mM sodium citrate and 36 μl of 30% H_2_O_2_, pH 4.0) was added to each well and incubated for 60 min. Then the plate was read using a microplate reader (Model 550, BioRad, California, USA) at 405–415 nm.

### Peptides

Peptides were synthesised at GL Biochem, Shanghai, China with more than 98% purity. The peptides contained the sequences as mentioned in Table 4.

**Table 4:** Peptides used in this study

**Name of the peptide**	**Sequence of the peptide**
L-P1 (Linear Peptide)	NDFRSKT

C-P1(Cyclic Peptide)	CNDFRSKTC

Control Peptide	CSWGEYDMC

### Cytotoxicity test by MTT assay

MDCK cells (~5000 cells/well) were grown on 96 well plates for 24 h. The media was replaced by serially diluted peptides or fusion phages and incubated again for 48 h. The culture medium was removed and 25 μl of MTT [3-(4,5-dimethylthiozol-2-yl)-3,5-dipheryl tetrazolium bromide] (Sigma) was added and incubated at 37°C for 5 h. Then 50 μl of DMSO was added to solubilised the formazan crystals and incubated for 30 mn. The optical density was measured at 540 nm in an microplate reader (Model 550, BioRad, USA).

### Virus yield reduction assay in egg allantoic fluid

The avian influenza A/Chicken/Iran/16/2000 (H9N2) virus suspension containing 8 or 16 HAU/50 μl was mixed with various concentrations of linear/cyclic peptides or fusion phages (50 μl) for 1 h at room temperature. This mixture was then injected into the allantoic cavity of 9 day-old embryonated chicken eggs and incubated at 37°C for 3 days. After incubation, the eggs were chilled for 5 h, the allantoic fluids were harvested and titrated by hemagglutination (HA) assay. As control, virus mixed with nonspecific peptides or wild phages were injected into the eggs.

### Hemagglutination inhibition assay

The hemagglutination inhibition (HI) assay was carried out as originally explained by Ramanujam et al., (2002) with slight modifications to evaluate the ability of the peptides/fusion phages to inhibit the viral adsorption to target cells. Linear/Cyclic peptides or fusion phages (50 μl) in serial two-fold dilutions in PBS were mixed with equal volume of influenza solution (8 HAU/50 μl) and incubated at room temperature for 1.5 h. Subsequently, 50 μl of 0.8% red blood cells were added to the above mixture and further incubated at room temperature for 45 min.

### Neuraminidase inhibition assay

The neuraminidase inhibition assay was carried out to test the ability of the peptide to inhibit the viral neuraminidase activity, as explained in Aymard-Hendry *et al*. [40] with slight modifications. The substrate used in this experiment was neuraminlactose rather than feutin.

### Preparation of Anti-AIV sera

Six month old New Zealand white rabbits were used for the production of polyclonal antibodies. Rabbits were pre-bleeded before injection. 50 μg of purified virus in PBS together with equal amount of Freund's adjuvant was injected into the rabbit subcutaneously. Subsequent booster injections were done with Freund's incomplete adjuvant. Injections were done for every 4 weeks, with bleeds 7 – 10 days after each injection. Antibodies were purified with Montage^® ^antibody purification kits (Millipore, USA) as instructed by the manufacturer.

### Antibody-Phage competition assay

Wells were coated with AIV subtype H9N2 (20 μg/ml; 100 μl) as the aforesaid conditions of biopanning. A mixture of purified polyclonal antibodies (1:500 dilutions; 100 μl) raised against AIV sub-type H9N2 and a series of different concentrations of phage FP-P1 (10^8 ^– 10^12 ^pfu; 100 μl) were prepared in eppendorf tubes. After blocking the wells, these mixtures were added and incubated at room temperature for 1 h. Wells were washed and bound phages were eluted and titrated. As for the positive control, AIV coated wells were incubated with the phage without the presence of the polyclonal antibodies.

### Peptide-Phage competition assay

The peptide – phage competition assay was performed to assay the inhibitory effects of synthetic peptides with its phage counterparts (FP-P1). AIV H9N2 was coated on a multi-well plate at the aforesaid conditions of biopanning and incubated with different concentrations of either linear of cyclic peptides (0.0001 – 1000 μM) in binding buffer for 1 h at 4°C. After 1 h incubation, phage FP-P1 (10^10 ^pfu/100 μl) was added and incubated at 4°C for another 1 h. Wells were then wash 6 times with TBST and the bound phages were eluted and titered. [Percentage of phage binding = (number of phage bound in the presence of peptide competitor/number of phage bound in the absence of peptide competitor) × 100].

### In vivo study of protein-protein interactions: Yeast two-hybrid assay

#### Cloning of HA_t_, NA and P1 genes into pYESTrp2 and pHybLex/Zeo vectors

The *NA *and truncated *HA *protein (*HA*_t_) genes of AIV sub-type H9N2 were amplified by Reverse Transcription-Polymerase Chain Reaction (RT-PCR) from the viral RNA using the primers pY-HA_t_-F & R and pY-NA-F & R, mentioned in Table 5. The *NA *gene carried the recognition sites for *Eco*RI and *Xho*I whereas the *HA*_t _gene carried the recognition sites *Kpn*I and *Xho*I restriction enzymes in their forward and reverse primers respectively. The peptide gene (*P1*) was amplified including the N1 domain of the P3 protein of the recombinant phage using the primer pH-P1-F & R (Table 5) from the ssDNA genome of the phage as the peptide is displayed as a fusion protein to this domain of the P3 protein. The *P1 *gene carried the recognition sites for *Eco*RI and *Xho*I restriction enzymes in its forward and reverse primers respectively. The amplified *HA*_t _and *NA *genes were ligated into pYESTrp2 vectors separately (Invitrogen, USA) and the *P1 *gene was cloned into pHybLex/Zeo (Invitrogen, USA) vector. The resultant clones were named as pY-HA, pY-NA and pH-P1 respectively. The constructs were sequenced using the primers pYESTrp2-F & R and pHybLex/Zeo-F & R (Table 5) to check the reading frame and for the absence of mutations. The *Saccharomyces cerevisiae *strain L40 was then co-transformed with the recombinant plasmids using lithium acetate method and the transformants were analysed for their β-galactosidase activity as explained in Ausubel *et al*. [41].

**Table 5:** Oligonucleotides used to amplify the *NA*, *HA*_t _and *P1*genes

**Primers**	**Sequence**
pY-NA-F^a^	5' CATAGAATTCGCAAAAGCAGGAGT 3'
pY-NA-R	5' TATCGCTCGAGAGTAGAAACAAGGAG 3'
pY-HA_t_-F	5' ATTTAAGGTACCGACAGCCATGGA 3'
pY-HAt-R	5' ATGCTGCTCGAGTATACAAATGTTGC 3'
pH-P1-F	5' AGCCTGGAATTCATGAAAAAATTA 3'
pH-P1-R	5' ATCGAACTCGAGATTTTCAGGGAT 3'
pHybLex/Zeo-F	5' AGGGCTGGCGGTTGGGGGTTATTCGC 3'
pHybLex/Zeo-R	5' GAGTCACTTTAAAATTTGTATACAC 3'
pYESTrp2-F	5' GATGTTAACGATACCAGCC 3'
pYESTrp2-R	5' GCGTGAATGTAAGCGTGAC 3'
pC-HA-F	5'ATTTAAGGATCCGAGAGCCATGGA 3'
pC-HA-R	5'ATGCTGCTCGAGTTATATACAAATGTTGC 3'
pC-NA-F	5'CATAGAATTCGCAAAAGCAGGAGT 3'
pC-NA-R	5'TATCGCTCGAGAGTAGAAACAAGGAG 3'
pC-P1-FP	5'AGCCTGGAATTCATGAAAAAATTA 3'
pC-P1-RP	5'CTCACTCGAGACATTTTCAGGGA 3'

^a^In all of the above mentioned oligonucleotides, the suffixes F and R refers Forward and Reverse primers respectively

### In vitro study of protein-protein interactions

#### Construction of recombinant pC-HA_t_, pC-NA and pC-P1 and in vitro transcription and translation

The *HA*_t _and *NA *gene of AIV strain H9N2 as well as the recombinant peptide gene *P1 *was amplified from pY-HA, pY-NA and pH-P1 respectively as templates using the primers pC-HA-F & R, pC-NA-F & R and pC-P1-F & R respectively (Table 5) and cloned into the pCITE2a vector. The in vitro transcription and translation was performed in a single tube in a reaction mixture (15 μl) containing circular recombinant plasmid (1 μg), TNT^® ^Quick Master Mix (12 μl; Promega, USA), Methionine (0.3 μl, 1 mM; Promega, USA). The above mixture was incubated at 30°C for 90 min. The translated products (3 μl) were electrophoresed on 15% SDS-PAGE and then transferred by electrophoresis for 1 h onto a nitrocellulose membrane. They were detected with anti-His antibody for P1 protein and HA_t_/NA proteins were detected with the polyclonal antibodies raised against the AIV sub-type H9N2 in rabbit.

#### Co-immunoprecipitation

Co-immunoprecipitation was performed using the Pierce^® ^Co-IP kit (Thermo Scientific, USA) as per the instructions given by the manufacturer. Briefly, the bait and pray complex was prepared separately by mixing the HA_t _or NA with His-conjugated P1 peptide. The complex was precipitated using purified anti-AIV polyclonal antibodies, which were immobilised on antibody coupling resin. The peptide P1 in the eluted co-immunoprecipitated complex was analysed by Western blotting using anti-His monoclonal antibodies (Novagen, USA) and detected with Amersham^® ^ECL^® ^western blotting detection reagents (GE Healthcare, USA).

### Statistical Analysis

All experiments were carried out in triplicate and are representative of at least three separate experiments. The results represent the means ± standard deviations or standard error means of triplicate determinations. Statistical significance of the data was determined by independent *t *test or one-way ANOVA method using SPSS software.

## Competing interests

MR is a graduate student of Universiti Putra Malaysia (UPM). FJ, ARO, AI and KY are employees of the same institution. The university holds the rights for all the financial benefits that may result from this research. Neither SSH nor her institution do not have any competing interests with this study. UPM is financing this manuscript as well. The UPM is the owner of the patent for the peptides mentioned in this manuscript (Patent No.: PI20082061).

## Authors' contributions

MR designed the study, carried out all of the experiments and drafted the manuscript. FJ participated in the design of yeast two hybrid assay experiments. ARO, AI, SSH participated in the design of avian influenza virus related experiments. KY conceived the study, participated in its design and co-ordination and helped to draft the manuscript. All authors read and approved the final manuscript.
